# Does kinesio taping plus exercise improve pain and function in patients with knee osteoarthritis?: A systematic review and meta-analysis of randomized controlled trials

**DOI:** 10.3389/fphys.2022.961264

**Published:** 2022-09-09

**Authors:** Haiyang Wu, Ruoyu Yao, Junhao Wu, Guowei Wen, Yiru Wang

**Affiliations:** ^1^ Longhua Hospital, Shanghai University of Traditional Chinese Medicine, Shanghai, China; ^2^ Huangpu Branch, Shanghai Ninth People’s Hospital, Shanghai Jiao Tong University School of Medicine, Shanghai, China

**Keywords:** kinesio taping, knee osteoarthritis, VAS, WOMAC, systematic review, meta-analysis

## Abstract

**Background:** Kinesio taping (KT) and exercise are described for improving pain and function of knee osteoarthritis (KOA) patients in most studies. However, the question remains if KT plus exercise is better than only exercise treatment.

**Objective:** To perform a systematic review and meta-analysis of randomized controlled trials (RCTs) to assess the effects of KT plus exercise in improving pain and knee function of KOA patients.

**Methods:** The databases PubMed, Cochrane Library, EMBASE, Springer, web of science and China National Knowledge Internet (CNKI) were searched till July 2022. People diagnosed with KOA were included. The intervention was KT plus exercise, but the comparison group was intervened only with exercise. Outcome measures were the Visual Analogue Scale (VAS) score, Western Ontario and McMaster Universities Osteoarthritis Index (WOMAC) score, and Timed Up and Go (TUG). Only RCTs were included. The Review Manager software (Version 5.3.5) was used to assess risk of bias, statistical heterogeneity and meta-analysis.

**Results:** The inclusion criteria were satisfied by 642 individuals from sixteen RCTs. There was a significant difference between KT plus exercise group and only exercise group in terms of VAS score after intervention (mean difference (MD) = −0.86; 95% CI = −1.32 to −0.40; *p* = 0.0003). In terms of VAS at follow-up period (MD = −0.58; 95% CI = −1.41 to 0.25; *p* = 0.17), WOMAC score (MD = 0.28; 95% CI = −9.16 to 9.71; *p* = 0.95) and TUG after intervention (MD = −0.74; 95% CI = −1.72 to 0.24; *p* = 0.14), no significant difference was found.

**Conclusion:** Although KT plus exercise reduced pain better than exercise, it did not enhance knee function in patients with KOA. These conclusions may change when more high-quality research is conducted.

## 1 Introduction

Knee osteoarthritis (KOA) is one of the most frequent types of osteoarthritis caused by repetitive motions of the knee joints. As the world’s elderly population grows, more people with KOA experience pain, edema, stiffness, and functional impairments (Jevsevar, 2013). This disease is accompanied with joint and muscle dysfunction, resulting in balance and gait difficulties (Thomas et al., 2009). KOA has risen to become the major cause of impairment in the elderly (Wang et al., 2012). According to current studies, roughly 18% of the elderly male and 27% of the elderly female suffer with KOA, and this ratio is expected to rise in the next decades (Krauss et al., 2016). As a result, KOA is less avoidable than is often supposed (Wallace et al., 2017).

The primary goals of therapy are to minimize or manage pain, enhance physical function and quality of life, and avoid disability. Severe KOA is best treated with unicompartmental or complete knee arthroplasties (Old et al., 2017). However, the surgical treatment’s success was frequently overestimated, while the procedure imposed a financial burden on patients. With the deterioration of physical function and willpower, the majority of the elderly experienced persistent pain and inadequate postoperative recovery (Peter et al., 2011). At the same time, pain may cause greater muscular weakness, leading in even more agony, with the process forming a vicious cycle of pain-weakness-pain. Based on these considerations, non-surgical therapy is the foundation of the 2020 guideline’s recommended method for treating KOA patients who don’t need surgery. Physical therapy, exercise therapy, and force line correction with health counseling and pharmacological treatment are examples of conservative therapies (Kolasinski et al., 2020). The aim is to improve mechan-transduction responses to influence articular metabolism and cartilaginous structure (Musumeci, 2016; Jorgensen et al., 2017). It is generally recommended to perform surgery only after conservative treatments have failed. In the case of those suffering from unbearable pain, surgery should be performed as soon as possible (Michael et al., 2010).

In addition to the conservative treatment options previously mentioned, the use of kinesio taping (KT) has become increasingly popular in patients with KOA. Dr. Kenzo Kase developed KT treatment in Japan in the 1970s. KT is commonly utilized in sports medicine and boosts players’ performance in the arena (Williams et al., 2012). KT may enhance muscular strength and knee-related status. It includes no medications and is effective in reducing edema, discomfort, and facilitating soft tissue function recovery (Abolhasani et al., 2019). KT is a kind of “myofiber” that exists outside the skin and pulls the skin while increasing the clearance between subcutaneous tissue and muscle. Increased local blood circulation and lymphatic circumfluence might alleviate swelling and discomfort (Campolo et al., 2013), reduce the strain on the muscles and repair the damaged soft tissue. KOA patients may benefit from KT because it relieves pain, reduces swelling, improves ligament function, increases range of motion, and stabilizes the knee joint (Campolo et al., 2013; Kalron and Bar-sela, 2013; Chao et al., 2016).

KT is progressively becoming acknowledged as a physical technique to treating KOA, and doctors and rehabilitation therapists are increasingly using it in clinical practice. KT has been shown to be effective for treating patellofemoral pain syndrome, coronal plane control and torsional control of the knee (Selfe et al., 2011). It has also been shown to increase isokinetic quadriceps torque (Anandkumar et al., 2014; Cho et al., 2015). However, a divisive position has been presented. Aytar et al. pointed out that KT application was not an effective treatment method for increasing joint position sense and reducing pain in patients with patellofemoral pain syndrome (Wageck et al., 2016). Additionally, the Lysholm Knee Scoring Scale and Western Ontario and McMaster Universities Osteoarthritis Index (WOMAC) score of elderly individuals who treated with KT treatment are not significantly different from those who with sham taping (Aytar et al., 2011). In contrast to Wageck et al. published in 2016, the study performed by Rahlf et al., in 2019 shows significant differences in the WOMAC score in patients with KOA between KT group and sham-KT group (Rahlf et al., 2019). Despite the fact that certain high-quality research on KT in KOA patients has been conducted, the conclusion as to whether KT has an impact or not remains equivocal. Based on a systematic review published in 2013, KT interventions are not recommended for these clinical populations with musculoskeletal conditions (Parreira Pdo et al., 2014). However, other three meta-analyses find that KT can reduce pain and enhance knee function when compare to sham KT (Lu et al., 2018; Ouyang et al., 2018; Melese et al., 2020). Lin et al. points out that KT versus physical therapy has substantial impact on pain and function alleviation in 2020 (Lin et al., 2020). Furthermore, according to the latest research, KT or KT combined with conventional therapy (physical therapy, rehabilitation, or medication) has a substantial impact on pain alleviation and isokinetic but not isometric muscular strength improvement in patients with KOA (Mao et al., 2021).

Appropriate exercise prescription is generally documented to be beneficial to KOA patients (Zeng et al., 2021). However, no review has yet been performed to compare KT plus exercise against exercise. Is KT with exercise more beneficial in reducing pain and improving knee function in those who have KOA? As a result, a meta-analysis of randomized controlled trials (RCTs) was conducted to verify the effectiveness of KT plus exercise in lowering pain and improving knee function in patients with knee OA.

## 2 Methods

The work was reported in line with PRISMA shown in Appendix 1 (Preferred Reporting Items for Systematic Reviews and Meta-Analyses) (Page et al., 2021) and registered in PROSPERO (registration identification: CRD42017060217; available website: https://www.crd.york.ac.uk/PROSPERO/#recordDetails).

### 2.1 Search strategy

Two reviewers (HYW and RYY) separately searched articles from the earliest date accessible to July 2022 using key phrases. By discussing the essential phrases proposed by the same two reviewers, only one search approach was validated. Electronic databases and manual searches were used to find articles. PubMed, CCTR, EMBASE, Springer, Web of Science, SinoMed, and China National Knowledge Internet (CNKI) were among the electronic databases used. To guarantee that all relevant papers were included, hand searching and professional journal reference lists were also performed. “Kinesio taping,” “knee osteoarthritis,” and “randomized controlled trial” were among the English phrases included in the search approach. There were no language or data restrictions on the articles. The two reviewers then used the eligibility criteria to find suitable studies through checking the titles and abstracts of all relevant papers. Finally, two reviewers rescreened complete texts of candidate articles using the same eligibility criteria to select the final articles that were included. Disagreements amongst the reviewers were resolved through discussion. If no agreement could be reached, a third reviewer (YRW) would decide whether or not the article should be included. Before being chosen, all of the reviewers had received training and had sufficient clinical expertise with the diagnosis and treatment of KOA. Appendix two shows the search strategy in detail.

### 2.2 Inclusion criteria

Inclusion criteria were confirmed by two independent reviewers (HYW and RYY). Studies were included if they matched the following PICOS criteria:

#### 2.2.1 Participants

Participants were adults who had been diagnosed with KOA by clinicians according to the American College of Rheumatology (ACR; formerly the American Rheumatism Association) (Altman et al., 1986; Belo et al., 2009) criteria, classified as grade 2 to 4 by the radiographicscale of Kellgren and Lawrence (Schiphof et al., 2008).

The ACR criteria of KOA are defined as follows:

- Clinical KOA is defined as knee pain and at least three out of six of the following criteria: age >50 years, morning stiffness <30 min, crepitus, bony tenderness, bony enlargement, and no palpable warmth.
- Clinical and radiographic KOA is defined as knee pain, osteophytes, and at least one out of three of the following criteria: age >50 years, morning stiffness <30 min, crepitus.
- Clinical and laboratory KOA is defined as knee pain and at least five out of nine of the following criteria: age >50 years, morning stiffness <30 min, crepitus, Erythrocyte Sedimentation Rate (ESR) < 40 mm/h, rheumatoid factor (RF) < 1:40, synovial fluid signs of OA (clear, viscous, or white blood cell count <2000/mm^3^).

#### 2.2.2 Intervention and comparision

The experimental groups were applied with KT therapy plus exercise. The controlled groups, on the other hand, were given exercise but no KT. Among the exercises performed were stretching of hamstrings and quadriceps muscles, alternating isometric and isotonic exercises for quadriceps, hip adductors, calf muscles, gluteus medius, and maximus, and open chain exercises, such as straight leg raises and leg raises with internal and external rotation, as well as closed chain exercises such as mini squats.

#### 2.2.3 Outcomes

The main outcome measure was Visual Analogue Scale (VAS) score after intervention. The secondary outcome measures were VAS score at follow-up period, WOMAC score and Timed Up and Go (TUG) after intervention. Due to the follow-up period, the KT intervention has been over for some time.

The VAS and WOAMC scores can be used to evaluate KOA symptoms and management. The validity and reliability of both examinations have been reported formerly (Basaran et al., 2010; Euasobhon et al., 2022). The VAS score, which is used to quickly classify symptom severity and disease control, is used to assess disease-related pain intensity (Shafshak and Elnemr, 2021). During the rest period, each patient is asked to report any discomfort they are experiencing. On a 0–10 numerical pain rating scale, zero indicates no pain and ten implies severe pain (Carlsson, 1983). The WOMAC score is quite helpful and commonly used in studies on knee health (Bellamy et al., 1988). The WOMAC score has 24 items and is divided into three categories: pain (5 items), stiffness (2 items), and physical function (17 items). The validity and reliability of TUG test have been described previously (Podsiadlo and Richardson, 1991). Patients sit in chairs at first and obey a clinician’s verbal direction, then stand and walk 3 m ahead, turn, and return to their seats. The duration of the entire test is reported in seconds.

#### 2.2.4 Study design

Randomized controlled trials (RCTs) were considered as potential included studies.

### 2.3 Exclusion criteria

Case reports, letters, basic experiments, self-controlled studies, non-randomised controlled trials or no exercise in controlled group were also excluded. Appendix three shows the excluded studies with reasons in detail.

### 2.4 Risk of bias and quality assessment

The risk of bias was assessed using Review Manager software (RevMan, Version 5.3.5, The Nordic Cochrane Centre, Copenhagen) and the 2011 revised Guidelines and Handbooks for Systematic Reviews in the Cochrane Back Review Group (Available from: http://community.cochrane.org). Each of the seven criteria was given a yes, no, or uncertain assessment. Random sequence generation (selection bias), allocation concealment (selection bias), blinding of participants and personnel (performance bias), blinding of outcome assessment (detection bias), incomplete outcome data (attrition bias), selective reporting (reporting bias), and other bias (such as wrong data, or unreasonable figures) were among the seven quality criteria. A study with a high risk was not be excluded, but it reduced our confidence in recommending this therapeutic strategy.

All included studies were examined regarding bias risk and methodological quality by two independent reviewers (HYW and GWW). A third party (JHW) was brought in to settle disagreements. Each of the three reviewers has previously worked in a training program.

### 2.5 Data extraction and analysis

Two independent reviewers (GWW and RYY) extracted relevant data from articles using a standardized form. Authors, publication date, number and demographics of participants, intervention of each group, period of follow-up, and results were among the data retrieved. To conduct this meta-analysis, all of the data was compiled into RevMan software. The I^2^ statistic was used to examine data homogeneity; if the value was greater than 50%, random-effect models were employed; otherwise, fixed-effect models were utilized (I^2^ > 50% was classed as moderate-to-high heterogeneity, and I^2^ ≤ 50% as low heterogeneity). The missing data were attempted to obtain by contacting the original author. The two independent reviewers had prior training expertise.

## 3 Results

### 3.1 Study selection

**FIGURE 1:**
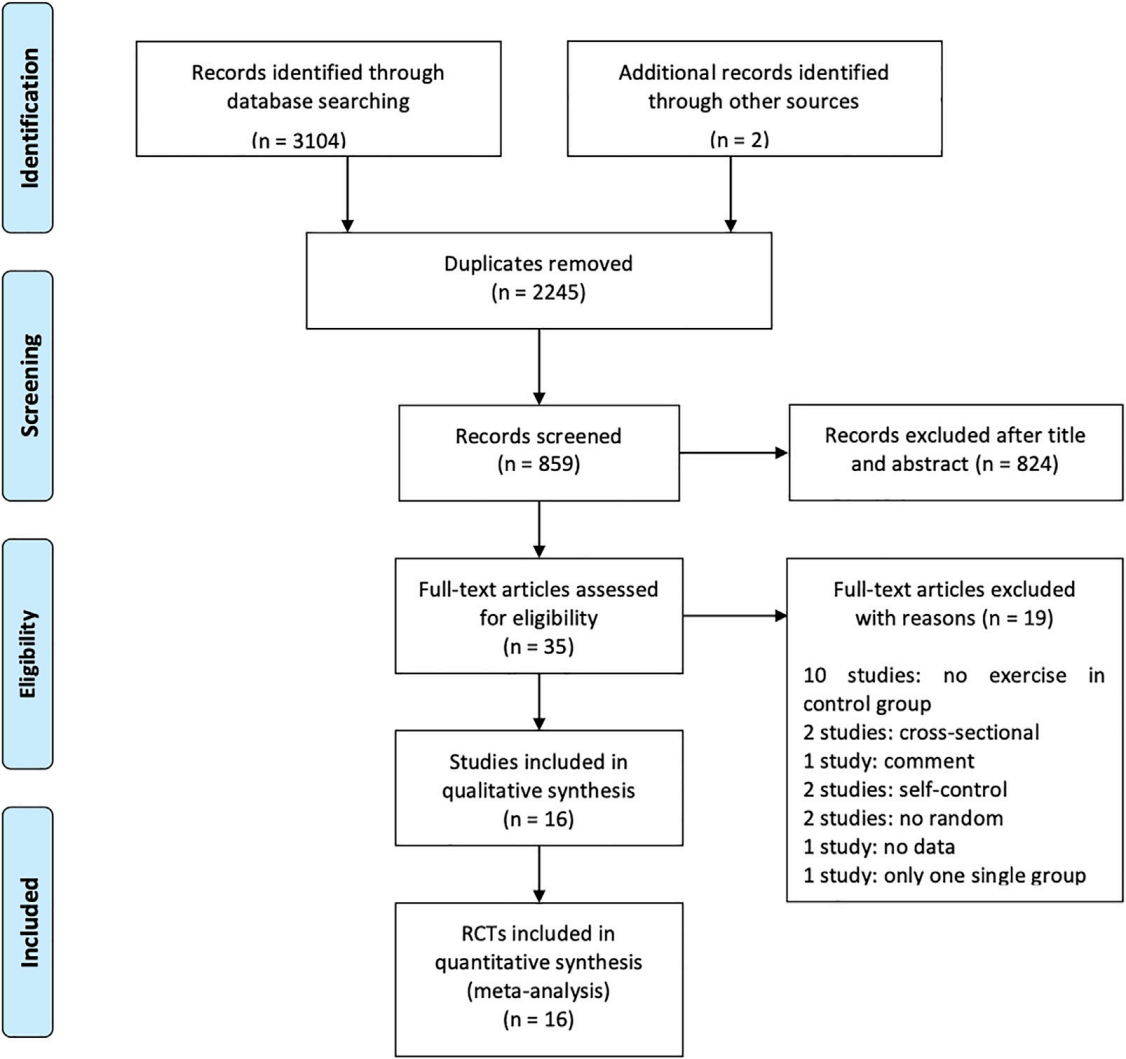
PRISMA flow diagram.

In the initial electronic database search and manual scanning, 859 studies were found (PubMed: 137, Cochrane Library: 170, EMBASE: 36, Springer: 1,249, Web of Science: 1,500, CNKI: 10, Hand searching and professional journals: 2) after removing 2,245 duplicates. The titles and abstracts of these were reviewed for inclusion and exclusion criteria, leaving 35 full articles. The 35 full text articles were evaluated in full texts according to the inclusion and exclusion criteria. Finally, 16 studies were included to perform the meta-analysis (Malgaonkar and Rizvi, 2014; Castrogiovanni et al., 2016; Dhanakotti et al., 2016; Sarallahi et al., 2016; Sedhom, 2016; Aydoğdu et al., 2017; Saswadkar et al., 2017; Taheri et al., 2017; Tripathi and Hande, 2017; Bao et al., 2018; Choi, 2018; Günaydin and Bayrakci Tunay, 2020; Leon-Ballesteros et al., 2020; Varma and Purohit, 2020; Danazumi et al., 2021; Oguz et al., 2021). In the excluded articles, no exercise was performed on ten studies, two were cross-sectional studies, one was a comment study, two were self-control studies, two studies were not randomized, one study had no data and one study had only one group (details were shown in Supplementary File S3). The PRISMA diagram flow is shown in Figure 1.

### 3.2 Description of studies

RCTs published in English, Chinese, or Korean between 2014 and 2021 were all included. Table 1 summarized the characteristics of the included studies. 16 studies involving 642 participants were analyzed, of which 323 were from the KT plus exercise group. The sample size ranged from 22 to 72, and the mean age of the patients was 48–64 years old. The intervention lasted from 3 days to 12 weeks. Each of the included studies described details about KT on knees, the frequency and duration of KT therapy, and exercise in the control group.

**TABLE 1:** Characteristics of the included studies.

Author	KT/CON				K-L (stage)	Intervention		Outcomes	At	MFs
	NP	Female	Age	BMI		KT group	CON group			
Aydoğdu 2017	28/26	ND	52.53/51.19	31.18/31.52	2 or 3	Y-shaped KT (10 and 5 cm) daily + exercise + hot pack + TENS for 6 weeks	exercise + hot pack + TENS	VAS, AROM	Week 6	no significant difference in VAS and AROM
Bao 2018	30/30	20/18	60.62/60.32	ND	ND	KTs (two Y-shaped + one X-shaped), each KT for 3–4 days, two times every week, for three times + exercise	exercise	VAS, WOMAC	Day 14, Day 30 (follow-up period)	significant difference in VAS and WOMAC
Castrogiovanni 2016	19/19	10/8	64.20/63.90	ND	2 or 3	KTs (two Y-strips in 20 cm with one I-strip) twice per week + exercise for 12 weeks	exercise	VAS, WOMAC, TUG	Day 15 and Week 12	significant difference in VAS and WOMAC
Choi 2018	12/12	12/12	67.91/67.54	ND	ND	5 cm kT for 6 weeks + exercise	exercise	VAS, quadriceps strength	Week 6	significant difference in all the outcomes
Danazumi 2021	30/30	ND	52.30/52.00	24.10/23.90	1 to 3	KTs (two Y-strips and one I-strip) + exercise, 3 treatment sessions per week for 8 weeks	exercise	VAS, AROM, TUG, SF-36	Week 8	significant difference in all the outcomes
Dhanakotti 2015	15/15	12/11	51.73/51.26	24.54/26.16	2 or 3	10 cm Y-strip and another 10 cm I-strip KT + exercise, 3 therapy sessions per week for total 3 weeks	exercise	VAS, WOMAC	Week 3	significant difference in VAS and WOMAC
Günaydin 2020	20/22	20/22	ND	ND	1 to 3	KTs (Y cut tape and two I bands) twice a week for 6 weeks + exercise for 12 weeks	only home exercise	VAS, TUG, 10mw, KOOS	Week 6, Week 12 (follow-up period)	significant difference in VAS, TUG, 10mw and KOOS
León-Ballesteros 2019	16/16	16/16	56.50/59.60	29.50/29.40	2 or 3	KTs (one I-shaped and one Y-shaped), once a week, for 6 weeks + exercise	exercise	VAS, WOMAC	Week 2, 4 and 6	no significant difference in VAS and WOMAC
Malgaonkar 2014	20/20	14/14	53.50/52.95	ND	3	KTs (Y-strip and I-strip) thrice a week + exercise for 2 weeks	exercise	VAS, WOMAC	Week 2	no significant difference in VAS and WOMAC
Oğuz 2021	11/11	11/11	48.18/51.00	30.90/34.76	2 or 3	KTs (I-shaped and Y-shaped) + exercise, 3 times per week, for 6 weeks	exercise	VAS, WOMAC, COMP, MMP-1, MMP-3	Week 6	significant difference in all the outcomes
Sarallahi 2018	19/19	19/19	55.63/55.63	ND	3 or 4	Y-shaped KT 3 days a week + exercise + hot pack + TENS for 3 weeks	exercise + hot pack + TENS	VAS, WOMAC, knee joint position sense	after 10 sessions	no significant difference in all the outcomes e
Saswadkar 2017	36/36	24/22	52.90/51.80	27.70/27.70	ND	Y strip kT with 25–30% tension + exercise for 3 days	exercise	VAS, gait parameters, stiffness, ADL	Day 3	significant difference in gait parameters, no significant difference in other outcomes
Sedhom 2016	20/20	20/20	49.25/48.70	ND	2 or 3	Y-shaped KTs + hot packs and exercise for 4 weeks; 3 sessions per week	hot packs and exercise	VAS, AROM, proprioceptive accuracy	Week 4	significant difference in all the outcomes
Taheri 2017	20/16	19/13	56.40/56.10	ND	2 or 3	KTs (two Y-shape and one I-shape) + exercise and medical therapy for 3 weeks	exercise and medical therapy	VAS, step test, TUG	Week 3, Week 6 (follow-up period)	no significant difference in all the outcomes
Tripathi 2016	15/15	ND	ND	ND	ND	KTs (Two Y-shaped), three times a week, for 3 weeks + exercise	exercise	VAS, WOMAC, TUG	Week 3	significant difference in all the outcomes
Varma 2017	12/12	ND	58.00/55.75	ND	2 or 3	Y-shaped KT (10 cm) + exercise, three section per week, for 2 weeks	exercise	VAS, WOMAC	Week 2	significant difference in VAS and WOMAC

KT, kinesio tape; CON, control group; NP, numbers of participants; BMI, body mass index; K-L, Kellgren-Lawrence radiographic criteria; AT, assess timing; ND, no data; TENS, transcutaneous electrical nerve stimulation; VAS, visual analogue scale; AROM, active range of motion; WOMAC, Western Ontario and McMaster Universities Arthritis Index; TUG, timed up and go; SF-36, short form-36 health survey; 10 mw, 10 m walk test; KOOS, knee injury and osteoarthritis outcome score.

In all included studies, the KTs were applied in different shapes with certain tension, such as Y-shape, I-shape and X-shape. A combination of two different shapes of KTs was used in nine studies, while only Y-shaped KT applied in six studies and no description in one study. The applied period of KT varied from 14 days to 6 weeks and frequency from daily to 4 days (details shown in Table 1).

### 3.3 Risk of bias and quality

**FIGURE 2:**
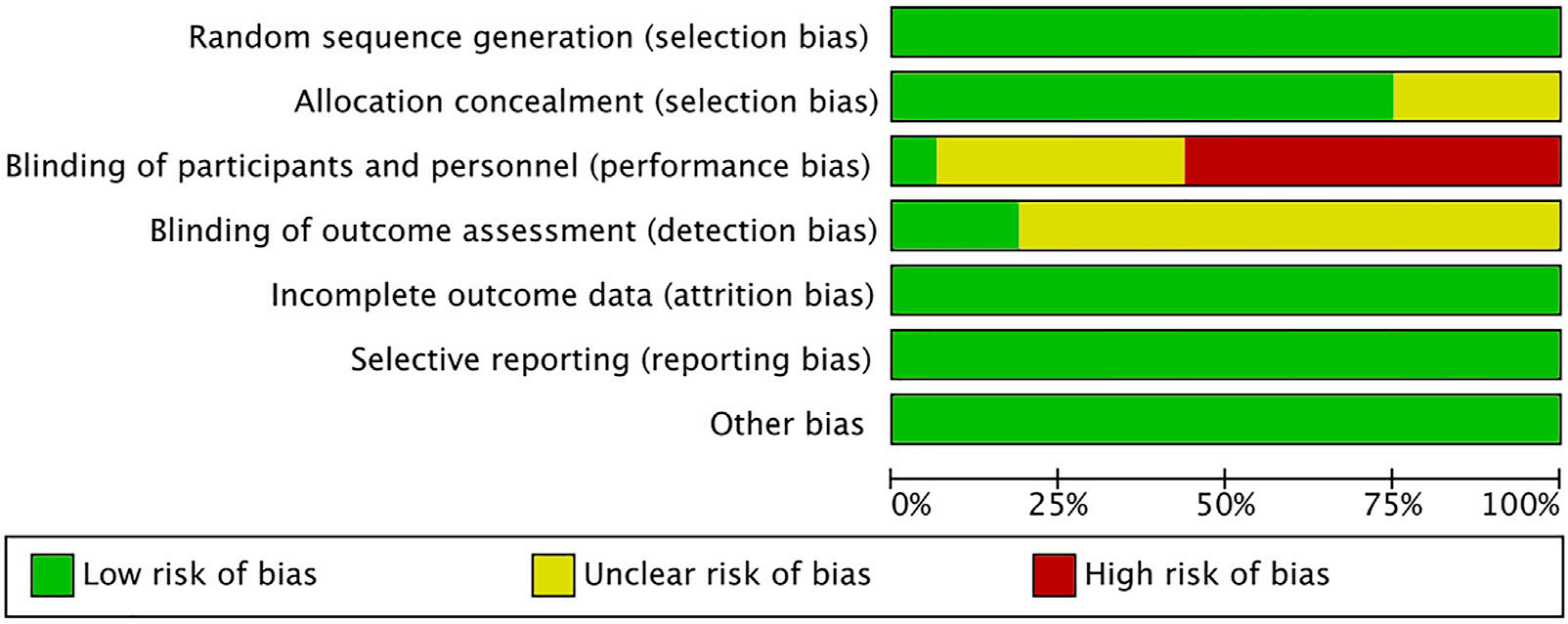
Risk of bias graph of included studies.

**FIGURE 3:**
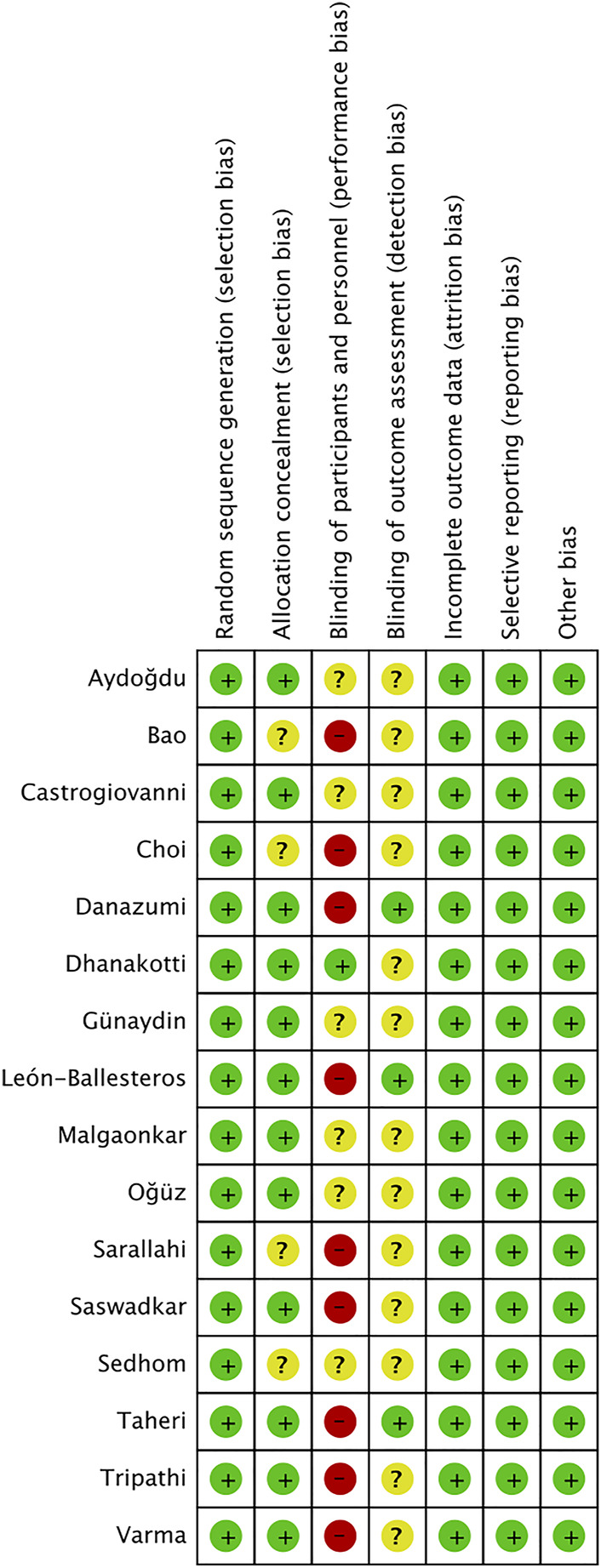
Risk of bias summary of included studies.

**FIGURE 4:**
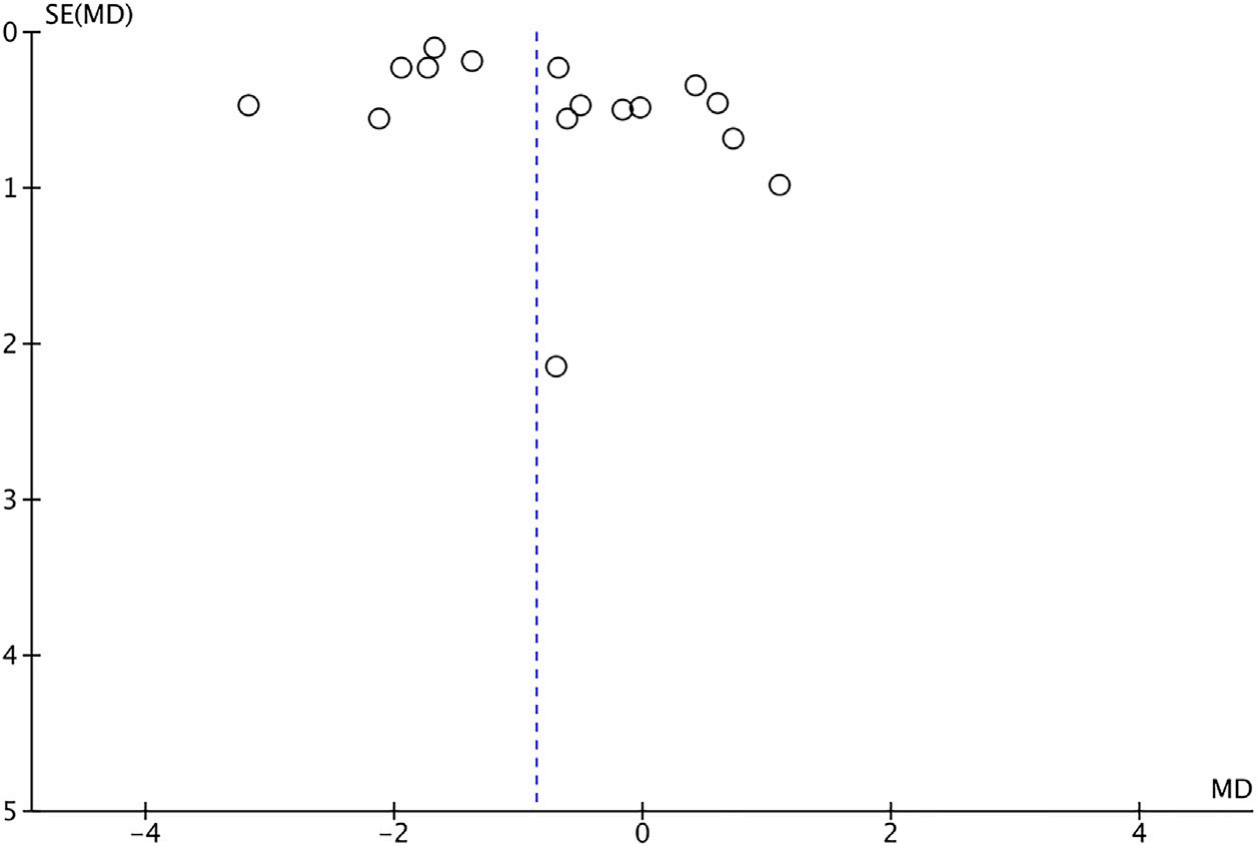
Risk of publication bias of included studies. SE, standard error; MD, mean deviation.

The risk of bias is shown in Figures 2, 3. Green areas means low risk of bias, yellow areas means unclear risk of bias, and the red areas represents high risk of bias. All studies were randomized and detailed random methods were described. Appropriate methods of allocation concealment were described except for four studies (Sarallahi et al., 2016; Sedhom, 2016; Bao et al., 2018; Choi, 2018). Blinding of participants and personnel was evaluated high risk in nine studies (Malgaonkar. et al., 2014; Castrogiovanni et al., 2016; Sedhom, 2016; Aydoğdu et al., 2017; Günaydin and Bayrakci Tunay, 2020; Oguz et al., 2021), low risk in one study (Dhanakotti et al., 2016) and unclear risk in other studies. Blinding of outcome assessments reached in three studies (Taheri et al., 2017; Leon-Ballesteros et al., 2020; Danazumi et al., 2021) and was not mentioned in other studies. Low risk of bias due to incomplete outcome data and selective outcome reporting was not detected in all the included studies. The VAS score funnel plot is symmetrical shown in Figure 4, indicating that there is a low risk of publication bias.

### 3.4 Outcomes and analysis

After intervention, sixteen RCTs (100%) investigated VAS scores and seven RCTs (43.75%) reported WOMAC scores. Interestingly, only three RCTs (18.75%) reported VAS scores at follow-up. In addition, TUG after intervention was observed in three RCTs (18.75%). All outcomes were analyzed using a random-effect model.

#### 3.4.1 VAS score after intervention

**FIGURE 5:**
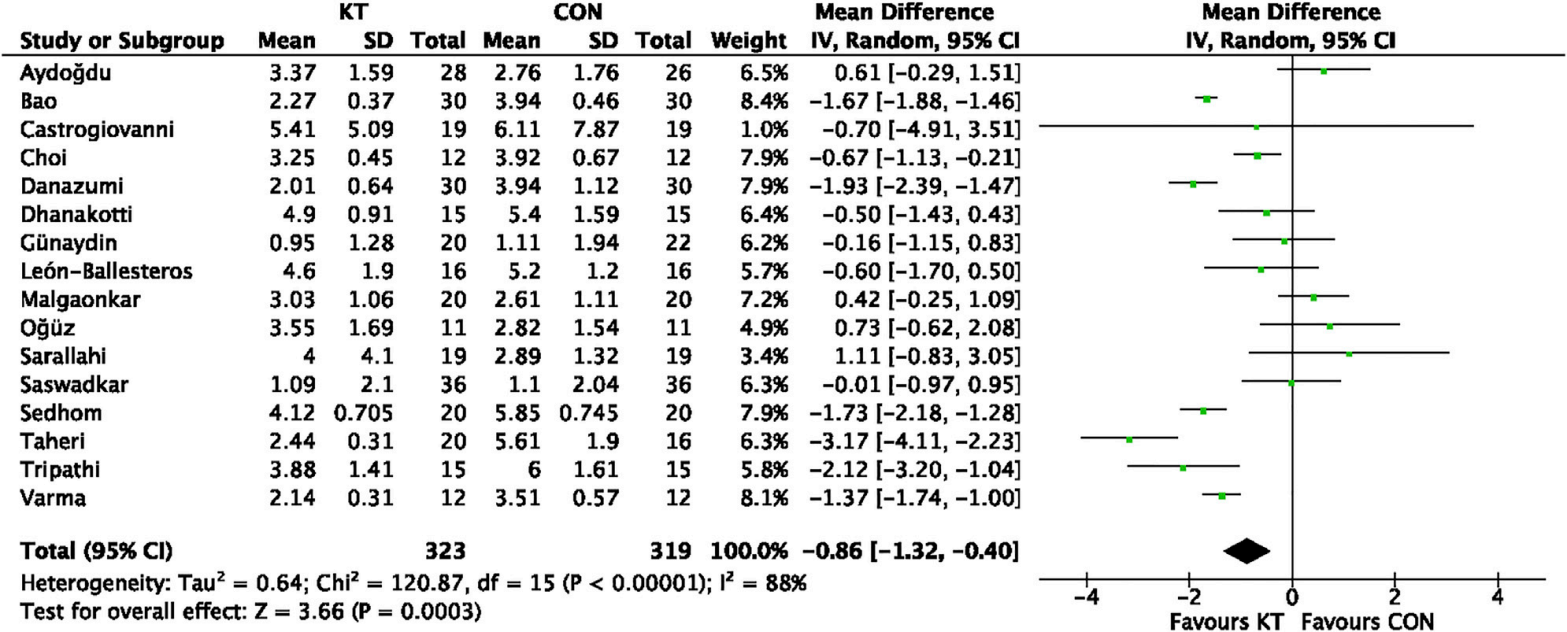
Forest plot of the meta-analyses comparing the KT group with control group for VAS score after intervention.

After intervention, VAS score were recorded in sixteen investigations. The analysis comprised a total of 642 participants. Because the pooled outcomes showed significant heterogeneity, this analysis used a random-effect model (χ^2^ = 120.87, I^2^ = 88%, *p* < 0.00001). The pooled data demonstrate a significant difference between the two groups (MD = −0.86; 95% CI = −1.32 to −0.40; *p* = 0.0003), as illustrated in Figure 5.

#### 3.4.2 VAS score at follow-up period

**FIGURE 6:**
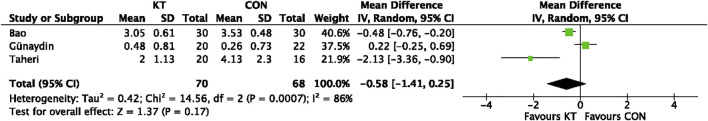
Forest plot of the meta-analyses comparing the KT group with control group for VAS score at follow-up period.

Four studies reported VAS scores during follow-up. The analysis comprised a total of 178 participants. Because the pooled outcomes showed significant heterogeneity, this analysis used a random-effect model (χ^2^ = 14.56, I^2^ = 86%, *p* = 0.0007). The pooled data demonstrate a significant difference between the two groups (MD = −0.58; 95% CI = −1.41 to 0.25; *p* = 0.17), as illustrated in Figure 6.

#### 3.4.3 WOMAC score after intervention

**FIGURE 7:**
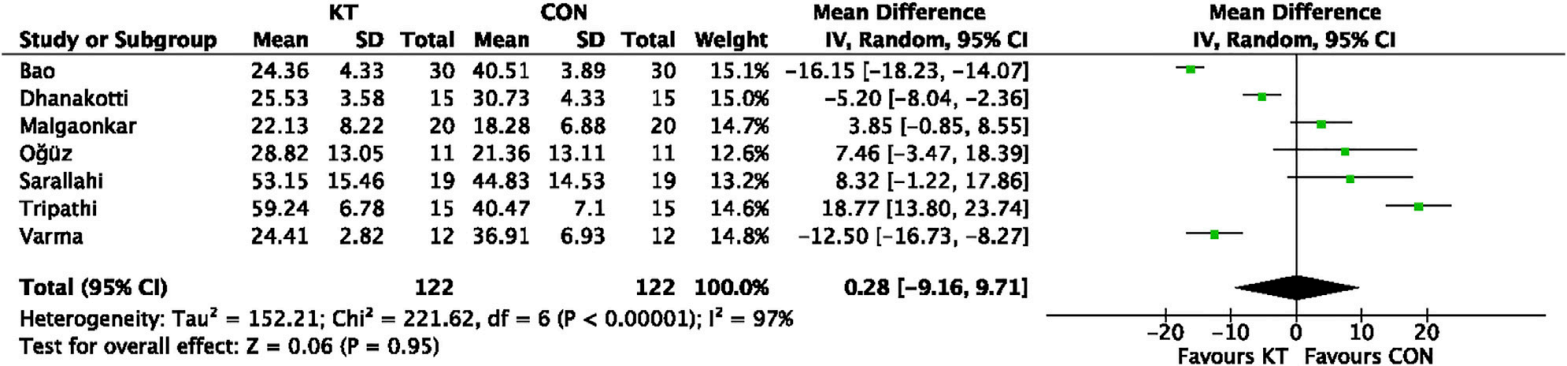
Forest plot of the meta-analyses comparing the KT group with control group for WOMAC score after intervention.

WOMAC score after intervention was reported in seven trials. The analysis comprised a total of 244 individuals. Because the pooled results showed significant heterogeneity, this investigation utilized a random-effect model (χ^2^ = 221.62 I^2^ = 97%, *p* < 0.00001). The pooled data show no significant difference between the two groups (MD = 0.28; 95% CI = −9.16 to 9.71; *p* = 0.95), as illustrated in Figure 7.

#### 3.4.4 TUG after intervention

**FIGURE 8:**
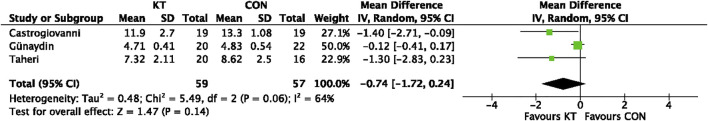
Forest plot of the meta-analyses comparing the KT group with control group for TUG score after intervention.

TUG score after intervention was reported in three trials. The analysis comprised a total of 116 individuals. Because the pooled results showed significant heterogeneity, this investigation utilized a random-effect model (χ^2^ = 5.49, I^2^ = 64%, *p* = 0.06). The pooled data show no significant difference between the two groups (MD = −0.74; 95% CI = −1.72 to 0.24; *p* = 0.14), as illustrated in Figure 8.

## 4 Discussion

Only RCTs of KOA patients treated with KTs plus exercise were included in this review and meta-analysis. On total, 642 individuals from sixteen RCTs were included in the meta-analysis. A meta-analysis was conducted using the collected data and revealed that KT plus exercise can significantly improve VAS score results. This indicated that KT plus exercise could help KOA sufferers experience less pain. However, KT plus exercise was unable to enhance knee function. Furthermore, data from included studies revealed that KT was not strongly connected to significant adverse outcomes.

The therapeutic hypothesis of KT, as derived from relevant investigations, consists mostly of the following recommendations. First of all, KT can improve proprioception by applying intrusive stimulation to the skin on a regular basis. Second, by strengthening weak muscles, physical function can be enhanced. Third, the elastic property of KT may be used to enlarge the subcutaneous area, allowing lymph and blood to circulate more freely. Fourth, by suppressing the nerve impulse, KT can lower pain intensity (Birmingham et al., 2001; Chang et al., 2010; Briem et al., 2011; O’Sullivan and Bird, 2011). However, these are not evidence-based statements, only theory-based.

The major proprioceptive receptors in knee joints are muscle and joint sensors (Birmingham et al., 2001). The periarticular and intraarticular receptors fail as a result of KOA pathological alterations. Consistent KT power can assist local soft tissues and strengthen or relax muscles depending on how it is used (Abolhasani et al., 2019). Firth et al., on the other hand, discover that KT treatment has no impact on the calf muscle. The lack of adequate afferent stimulation in boosting the function of healthy muscle, or variances in tape procedures and materials, might be the cause. In treating weak muscle in non-athletes and patients with KOA, positive benefits should be applauded (Firth et al., 2010). The accumulation of pain signals can be reduced *via* lymph and blood circulation. Meanwhile, the tactile afferent neuron has a bigger diameter than the algetic afferent nerve (Campolo et al., 2013). However, some researchers point that placebo effect might attribute to the positive findings of KTs (Lumbroso et al., 2014; Mak et al., 2019). The potential adverse effects are delayed treatment and increased patient distress.

The change in VAS score is used as the primary indicator of pain decrease. Pain is reduced just after KT plus exercise therapy, but no change during the follow-up period. The duration of follow-up was defined as a period of time after the KT intervention has ended before VAS scores are measured. Only three studies report this outcome (Taheri et al., 2017; Bao et al., 2018; Günaydin and Bayrakci Tunay, 2020). Van der et al. conduct a 3-years prospective cohort analysis of 146 individuals with early KOA, finding that periarticular muscular weakness is linked to daily activity restriction and discomfort (Van der Esch et al., 2014). The loss in motor neuron might be compensated by using taping methods with no draw force or by placing the KT in specific body areas with a lot of sensory receptors. The enhancement of afferent nerve impulses from the ligament, skin, and capsule of the knee-joint helps to prevent quadriceps muscle weakness induced by Ia afferent activity (Konishi, 2013). Furthermore, four other systematic reviews come to the same conclusion as the present study (Lu et al., 2018; Ouyang et al., 2018; Lin et al., 2020; Mao et al., 2021).

The WOMAC score is used to determine the health of the knees. The three main domains of the WOAMC questionnaire are pain, joint stiffness, and physical activities. The overall score is calculated by adding the three domain scores and ranges from 0 to 96; a high score indicates poor health. KT stimulates mechanoreceptors, which can accurately convey articular position signals during activities. It is thought to be a valid rationale for KT in terms of increasing joint range of motion (Yoshida and Kahanov, 2007). WOMAC scores are lowered after KTs intervention in two previous investigations (Lu et al., 2018; Lin et al., 2020). However, our findings revealed that KT had no impacts on knee-related physical function in KOA patients. The TUG result is also utilized to evaluate knee function. There was a substantial difference in TUG outcome between the two groups in our study. It was in line with a previous review (Ouyang et al., 2018).

To our knowledge, this is the most recent review and meta-analysis comparing the efficacy of KT plus exercise therapy vs. merely active exercise in treating KOA. New studies were included that had not been analyzed in previous reviews. The conclusion was also revised because new studies were added. While, there were also some potential limitations. We did our best to find relevant publications using various methods without limitations of language, database or publication time. However, some studies only published in papers in their local countries, or some negative results were not published. It was failed to obtain all these data. As a result, bias of selection could not be avoided. Across all studies included, participants were from a variety of countries and ages, held a wide variety of occupations, and even belonged to different Kellgren-Lawrence radiographic levels. The therapists applied the KT with various shapes, tensions, directions, lengths and intervention periods. It was difficult to avoid a high heterogeneity (Aytar et al., 2011).

## 5 Conclusion

The results revealed that KT plus exercise had a positive and beneficial effect on pain reduction when compared to merely exercise, but that it had a negative influence on knee function improvement. In the future, new high-quality and longer intervention period research may modify these figures.

## Data Availability

The original contributions presented in the study are included in the article/Supplementary Material, further inquiries can be directed to the corresponding author.
