# Supplementary material for: Does kinesio taping plus exercise improve pain and function in patients with knee osteoarthritis?: A systematic review and meta-analysis of randomized controlled trials
Source: Front Physiol. 2022 Sep 9;13:961264. doi: 10.3389/fphys.2022.961264 (PMC9500481; doi:10.3389/fphys.2022.961264)
Supplement: Supplementary file 1 [file DataSheet2.PDF]

Search strategy in Pubmed database

1 Knee Osteoarthritis [mh]

2 (Knee Osteoarthritides or Knee Osteoarthritis or Osteoarthritides, Knee or Osteoarthritis of Knee or Knee, Osteoarthritis Of or Knees, Osteoarthritis Of or Osteoarthritis Of Knees) [tw]

3 1 or 2

4 osteoarthritis [mh]

5 (Osteoarthritides or Osteoarthritis or Osteoarthroses or Arthritis, Degenerative or Arthritides, Degenerative or Degenerative Arthritides or Degenerative Arthritis or Osteoarthrosis Deformans) [tw]

6 4 or 5

7 knee [mh]

8 knee joint [mh]

9 (Joint, Knee or Joints, Knee or Knee Joints or Superior Tibiofibular Joint or Joint, Superior Tibiofibular or Joints, Superior Tibiofibular or Superior Tibiofibular Joints or Tibiofibular Joint, Superior or Tibiofibular Joints or Superior) [tw]

10 7 or 8 or 9

11 6 and 10

12 3 or 11

13 Kinesio taping [mh]

14 (Kinesio Tapes or Tape, Kinesio or Tapes, Kinesio or Skin Tape or Skin Tapes or Tape, Skin or Tapes, Skin or Adhesive Kinesio Tape or Adhesive Kinesio Tapes or Kinesio Tape, Adhesive or Kinesio Tapes, Adhesive or Tape, Adhesive Kinesio or Tapes, Adhesive Kinesio or Adhesive Tape, Kinesio or Adhesive Tapes, Kinesio or Kinesio Adhesive Tape or Kinesio Adhesive Tapes or Tape, Kinesio Adhesive or Tapes, Kinesio Adhesive) [tw]

15 13 or 14

16 randomized controlled trial [pt]

17 controlled clinical trial [pt]

18 randomized [tiab]

19 palcebo [tiab]

20 human trials as topic [mesh: noexp]

21 randomly [tiab]

22 trial [ti]

23 16 or 17 or 18 or 19 or 20 or 21 or 22

24 humans [mh] NOT animals [mh]

25 23 and 24

26 12 and 15 and 25

PubMed search syntax

[mh] denotes a Medical Subject Heading (Mesh) term (‘exploded’);

[tw] denotes text word;

[pt] denotes a Publication Type term;

[tiab] denotes a word in the title or abstract;

[sh] denotes a subheading;

[mesh: noexp] denotes a Medical Subject Heading (Mesh) term (not ‘exploded’);

[ti] denotes a word in the title.

Search strategy for Cochrane Library database

#1 MeSH descriptor: [knee osteoarthritis] explode all trees

#2 MeSH descriptor: [osteoarthritis] explode all trees

#3 (knee arthritis):ti,ab,kw

#4 (knee pain):ti,ab,kw

#5 (knee damage):ti,ab,kw

#6 (knee swell):ti,ab,kw

#7 (knee weakness):ti,ab,kw

#8 #1 or #2 or #3 or #4 or #5 or #6 or #7

#9 MeSH descriptor: [Exercise] explode all trees

#10 MeSH descriptor: [Sports] explode all trees

#11 #9 or #10

#12 MeSH descriptor: [Kinesio taping] explode all trees

#13 #8 and #11 and #12

Cochrane Library search syntax

[ti] denotes a word in the title.

[ab] denotes a word in the abstract.

[kw] denotes a word in the keywords.

Search strategy for EMBASE

#1 'knee osteoarthritis' /exp

#2 'osteoarthritis' /exp

#3 'knee arthritis':ab,ti

#4 'knee pain':ab,ti

#5 'knee damage':ab,ti

#6 'knee swell':ab,ti

#7 'knee weakness':ab,ti

#8 #1 OR #2 OR #3 OR #4 OR #5 OR #6 OR #7

#9 'exercise' /exp

#10 'sports' /exp

#11 #9 or #10

#12 'Kinesio taping' /exp

#13 'randomized controlled trial' /exp

#14 #8 and #11 and #12 and #13

EMBASE search syntax

[exp] denotes explosion.

[ab] denotes a word in the abstract.

[ti] denotes a word in the article title.

Search Strategy for Web of science:

#1 TS= (exercise OR sports)

#2 TS= (injuries OR injury OR tenderness OR pain OR myalgia OR soreness OR damage OR fatigue OR weakness)

#3 TS= muscle

#4 #1 AND #2 AND #3

#5 TS= (Randomized Controlled Trials OR trial OR placebo OR groups OR control OR controlled OR Random*)

#6 TS= vibration

#7 #4 and #5 and #6

Search Strategy for CNKI

FT=随机+对照+随机对照+随机对照研究+随机对照试验+随机分配 AND SU=肌内效贴 AND SU=膝骨关节炎+骨关节炎+膝关节炎+膝痛+KOA
