# Supplementary material for: Does kinesio taping plus exercise improve pain and function in patients with knee osteoarthritis?: A systematic review and meta-analysis of randomized controlled trials
Source: Front Physiol. 2022 Sep 9;13:961264. doi: 10.3389/fphys.2022.961264 (PMC9500481; doi:10.3389/fphys.2022.961264)
Supplement: Supplementary file 2 [file DataSheet3.PDF]

Total 19 excluded studies with reasons

- 10 studies: no exercise in control group (Anandkumar et al., 2014; Cho et al., 2015; Kocyigit et al., 2015; Kaya Mutlu et al., 2016; Ogut et al., 2018; Abolhasani et al., 2019; Donec and Kubilius, 2019; Farhadian et al., 2019; Hayati et al., 2019; Pinheiro et al., 2020)
- 2 studies: cross-sectional (Hinman et al., 2003; Gomes et al., 2018)
- 1 study: comment (Hinman et al., 2004)
- 2 studies: self-control (Cushnaghan et al., 1994; Tani et al., 2018)
- 2 studies: no random (Edmonds et al., 2016; Lee et al., 2016)
- 1 study: no data (Ibrahim and Atya, 2013)
- 1 study: only one single group (Park et al., 2019)

Reference

Abolhasani, M., Halabchi, F., Honarpishe, R., Cleland, J.A., and Hakakzadeh, A. (2019). Effects of kinesiotape on pain, range of motion, and functional status in patients with osteoarthritis: a randomized controlled trial. *J Exerc Rehabil* 15(4), 603-609. doi: 10.12965/jer.1938290.145.

Anandkumar, S., Sudarshan, S., and Nagpal, P. (2014). Efficacy of kinesio taping on isokinetic quadriceps torque in knee osteoarthritis: a double blinded randomized controlled study. *Physiother Theory Pract* 30(6), 375-383. doi: 10.3109/09593985.2014.896963.

Cho, H.Y., Kim, E.H., Kim, J., and Yoon, Y.W. (2015). Kinesio taping improves pain, range of motion, and proprioception in older patients with knee osteoarthritis: a randomized controlled trial. *Am J Phys Med Rehabil* 94(3), 192-200. doi: 10.1097/PHM.0000000000000148.

Cushnaghan, J., McCarthy, C., and Dieppe, P. (1994). Taping the patella medially: a new treatment for osteoarthritis of the knee joint? *BMJ* 308(6931), 753-755. doi: 10.1136/bmj.308.6931.753.

Donec, V., and Kubilius, R. (2019). The effectiveness of Kinesio Taping(R) for pain management in knee osteoarthritis: a randomized, double-blind, controlled clinical trial. *Ther Adv Musculoskelet Dis* 11, 1759720X19869135. doi: 10.1177/1759720X19869135.

Edmonds, D.W., McConnell, J., Ebert, J.R., Ackland, T.R., and Donnelly, C.J. (2016). Biomechanical, neuromuscular and knee pain effects following therapeutic knee taping among patients with knee osteoarthritis during walking gait. *Clin Biomech (Bristol, Avon)* 39, 38-43. doi: 10.1016/j.clinbiomech.2016.09.003.

Farhadian, M., Morovati, Z., and Shamsoddini, A. (2019). Effect of Kinesio Taping on Pain, Range of Motion, Hand Strength, and Functional Abilities in Patients with Hand Osteoarthritis: A Pilot Randomized Clinical Trial. *Arch Bone Jt Surg* 7(6), 551-560.

Gomes, C., Dibai-Filho, A.V., Biasotto-Gonzalez, D.A., Politti, F., and Camillo de Carvalho, P.T. (2018). Association of Pain Catastrophizing With Static Balance, Mobility, or Functional Capacity in Patients With Knee Osteoarthritis: A Blind Cross-sectional Study. *J Manipulative Physiol Ther* 41(1), 42-46. doi: 10.1016/j.jmpt.2017.08.002.

Hayati, M., Yazdi, Z., and Abbasi, M. (2019). Comparison of non-steroidal anti-inflammatory drugs and knee kinesio taping in early osteoarthritis pain: A randomized controlled trial. *J Bodyw Mov Ther* 23(3)**,** 666-670. doi: 10.1016/j.jbmt.2018.06.011.

Hinman, R., Crossley, K., McConnell, J., and Bennell, K. (2004). Therapeutic knee taping improved pain and disability in osteoarthritis of the knee. *ACP J Club* 140(1)**,** 17.

Hinman, R.S., Crossley, K.M., McConnell, J., and Bennell, K.L. (2003). Efficacy of knee tape in the management of osteoarthritis of the knee: blinded randomised controlled trial. *BMJ* 327(7407)**,** 135. doi: 10.1136/bmj.327.7407.135.

Ibrahim, A.R., and Atya, A. (2013). "Kinesio Taping Versus Sensorymotor Training For Patients With Knee Osteoarthritis", in: *International Journal of Therapies and Rehabilitation Research.*).

Kaya Mutlu, E., Mustafaoglu, R., Birinci, T., and Razak Ozdincler, A. (2016). Does Kinesio Taping of the Knee Improve Pain and Functionality in Patients with Knee Osteoarthritis?: A Randomized Controlled Clinical Trial. *Am J Phys Med Rehabil*. doi: 10.1097/PHM.0000000000000520.

Kocyigit, F., Turkmen, M.B., Acar, M., Guldane, N., Kose, T., Kuyucu, E., et al. (2015). Kinesio taping or sham taping in knee osteoarthritis? A randomized, double-blind, sham-controlled trial. *Complement Ther Clin Pract* 21(4)**,** 262-267. doi: 10.1016/j.ctcp.2015.10.001.

Lee, K., Yi, C.W., and Lee, S. (2016). The effects of kinesiology taping therapy on degenerative knee arthritis patients' pain, function, and joint range of motion. *J Phys Ther Sci* 28(1)**,** 63-66. doi: 10.1589/jpts.28.63.

Ogut, H., Guler, H., Yildizgoren, M.T., Velioglu, O., and Turhanoglu, A.D. (2018). Does Kinesiology Taping Improve Muscle Strength and Function in Knee Osteoarthritis? A Single-Blind, Randomized and Controlled Study. *Arch Rheumatol* 33(3)**,** 335-343. doi: 10.5606/ArchRheumatol.2018.6598.

Park, J.S., Yoon, T., Lee, S.H., Hwang, N.K., Lee, J.H., Jung, Y.J., et al. (2019). Immediate effects of kinesiology tape on the pain and gait function in older adults with knee osteoarthritis. *Medicine (Baltimore)* 98(45)**,** e17880. doi: 10.1097/MD.0000000000017880.

Pinheiro, Y.T., Barbosa, G.M., Fialho, H.R.F., Silva, C.A.M., Anunciacao, J.O., Silva, H.J.A., et al. (2020). Does tension applied in kinesio taping affect pain or function in older women with knee osteoarthritis? A randomised controlled trial. *BMJ Open* 10(12)**,** e041121. doi: 10.1136/bmjopen-2020-041121.

Tani, K., Kola, I., Shpata, V., and Dhamaj, F. (2018). Evaluation of Gait Speed after Applying Kinesio Tape on Quadriceps Femoris Muscle in Patients with Knee Osteoarthritis. *Open Access Maced J Med Sci* 6(8)**,** 1394-1398. doi: 10.3889/oamjms.2018.273.
